# *PgDDS* Changes the Plant Growth of Transgenic *Aralia elata* and Improves the Production of Re and Rg_3_ in Its Leaves

**DOI:** 10.3390/ijms25031945

**Published:** 2024-02-05

**Authors:** Wenhua Guo, Yue Zhao, Honghao Xu, Yuxin Xia, Lei Tao, Xiangling You

**Affiliations:** Key Laboratory of Saline-Alkali Vegetation Ecology Restoration, Ministry of Education, Northeast Forestry University, Harbin 150040, China; guowenhua@nefu.edu.cn (W.G.); zhaoyuezy@nefu.edu.cn (Y.Z.); xhhnefu@163.com (H.X.); 19917627531@163.com (Y.X.); taolei2003-01@163.com (L.T.)

**Keywords:** *Aralia elata*, tetracyclic triterpenoid, pentacyclic triterpenoid, terpene metabolome

## Abstract

*Aralia elata* (Miq.) Seem is a medicinal plant that shares a common pathway for the biosynthesis of triterpenoid saponins with *Panax ginseng*. Here, we transferred the dammarenediol-II synthase gene from *P. ginseng* (*PgDDS*; GenBank: AB122080.1) to *A. elata*. The growth of 2-year-old transgenic plants (L27; 9.63 cm) was significantly decreased compared with wild-type plants (WT; 74.97 cm), and the leaflet shapes and sizes of the transgenic plants differed from those of the WT plants. Based on a terpene metabolome analysis of leaf extracts from WT, L13, and L27 plants, a new structural skeleton for ursane-type triterpenoid saponins was identified. Six upregulated differentially accumulated metabolites (DAMs) were detected, and the average levels of Rg3 and Re in the leaves of the L27 plants were 42.64 and 386.81 μg/g, respectively, increased significantly compared with the WT plants (15.48 and 316.96 μg/g, respectively). Thus, the expression of *PgDDS* in *A. elata* improved its medicinal value.

## 1. Introduction

*Aralia elata* (Miq.) Seem (Araliaceae) is a species of deciduous woody shrub or small tree that is mainly distributed in Northeast China, Korea, and Russia [1]. The leaves, bark, and roots of *A. elata* are important Chinese traditional folk medicines [2] that are used to treat diseases such as hepatitis, diabetes, and gastrospasm [3] and have anti-inflammatory and cardio-protective activities [4]. To date, more than 100 kinds of simple saponins have been isolated from *A. elata* [5], and their specific functions are being revealed [3]. In addition to saponins, the buds also contain a variety of nutrients needed by the human body, and there is a large market for *A. elata* in South Korea, Japan, and China. In Heilongjiang Province in Northeast China, the annual economic benefits of the buds are considerable; for example, the quarterly sale volume was 10^6^ Kg in 2010 in Helong, Heilongjiang Province [6].

**Figure 1:**
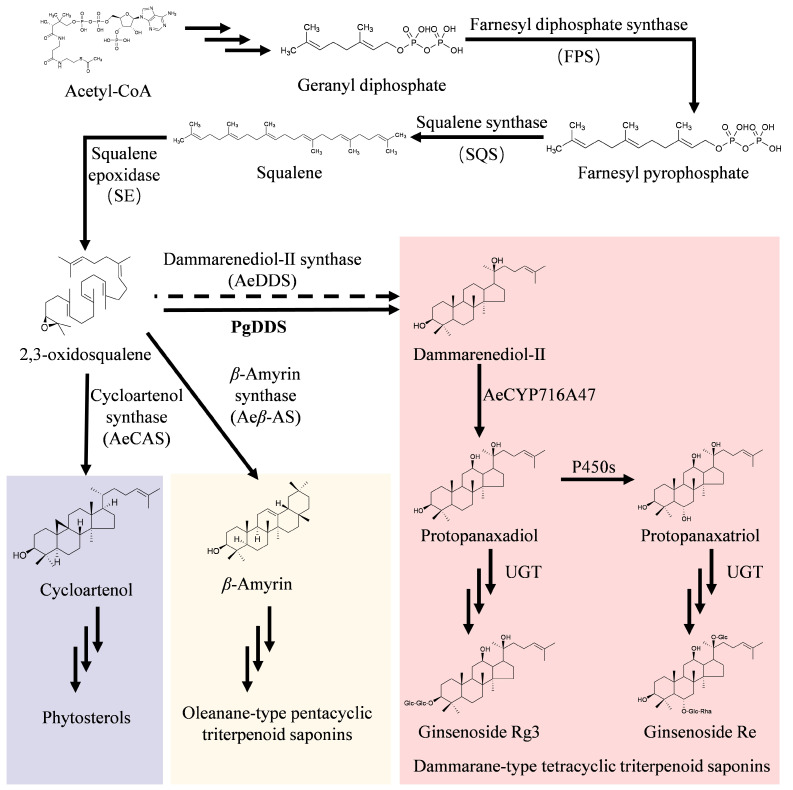
The triterpenoid saponin biosynthesis pathway in *Aralia elata*. The dashed arrow indicates that a gene encoding the *A. elata* dammarenediol-II synthase (*Ae*DDS) enzyme could not be detected. *PgDDS* is the DDS enzyme from *Panax ginseng* expressed in *A. elata*. Purple square, phytosterols; Yellow square, oleanane-type pentacyclic triterpenoid saponins; red square, dammarane-type tetracyclic triterpenoid saponins.

The other medicinal species in the family Araliaceae, *Panax ginseng*, is a well-known traditional Chinese medicine in China [7]. Ginseng also contains two kinds of saponins, pentacyclic triterpenoid saponins and tetracyclic triterpenoids (Figure 1). Pentacyclic triterpenoid saponins are the main active components in *A. elata*, although a few tetracyclic triterpenoids (ginsenosides Re) are also present [8,9]. The proportion of saponins in *P. ginseng* is the opposite of that in *A. elata*; the main saponins are tetracyclic triterpenoid saponins, and the proportion of pentacyclic triterpenoid saponins is very low [10,11]. This difference is mainly caused by the content and expression of two kinds of cyclases in the saponin biosynthesis pathway. The main cyclase in *A. elata* is β-amyrin synthase (β-AS), while the main cyclase in *P. ginseng* is dammarenediol-II synthase (PgDDS) [11]. Chun et al. [12] transformed *PgDDS* and the downstream gene *CYP716A47* that encodes the protopanaxadiol synthase gene into tobacco and obtained medicinal dammarane saponins in the transformed tobacco cell lines. In the same year, another group transformed rice with the ginseng *PgDDS* gene and produced protopanaxadiol in rice, making rice into a functional food [13]. However, the other effects of *PgDDS* gene expression on the transgenic tobacco and rice lines, such as growth rate, relative key enzyme gene expression, and changes in endogenous saponins, were not determined.

In this study, due to the importance of *A. elata* as a medicinal product and edible plant in Heilongjiang Province, our objective was to transform *A. elata* with the *PgDDS* gene in an effort to change its saponin components and potentially increase its medicinal and edible qualities. We also looked at (1) the effects of *PgDDS* gene expression on the growth of the transgenic plants, (2) changes in the expression of key enzyme genes associated with saponin synthesis such as farnesyl diphosphate synthase (*FPS*), squalene synthase (*SQS*), squalene epoxidase (*SE*), *β-AS*, cycloartenol synthase (*CAS*), and *AeCYP716A47* (Figure 1), and (3) the saponin content and types of pentacyclic triterpenoids and tetracyclic triterpenoids in the leaves of transgenic plants.

## 2. Results

### 2.1. Genetic Transformation and Heterologous PgDDS Expression in A. elata

**Figure 2:**
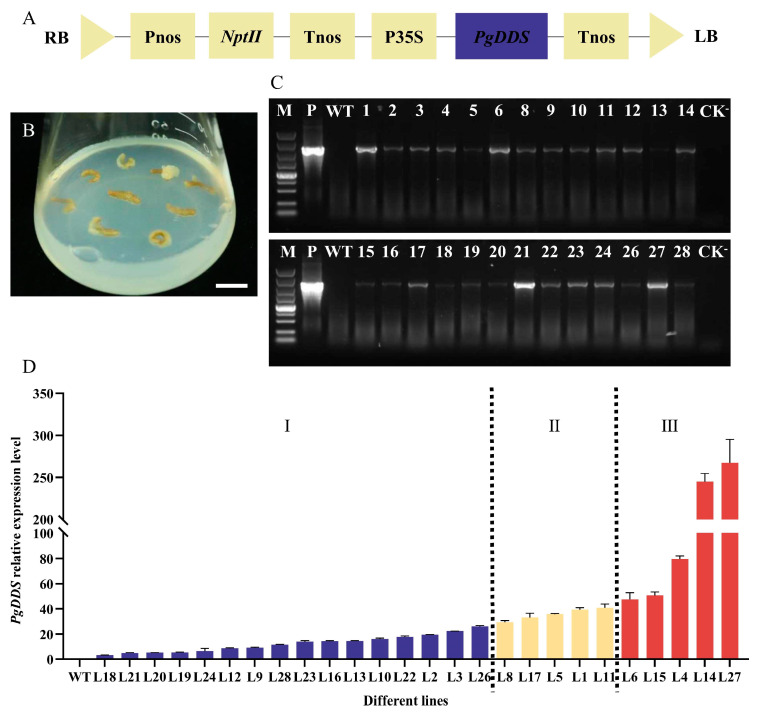
Detection of *PgDDS* and its expression in the calli of transgenic *A. elata*. (**A**) Schematic diagram showing the construction of the plant overexpression vector pROKII-*PgDDS*. LB, T-region left border; Pnos, nopaline synthase promoter; *NptII*, kanamycin resistance gene; Tnos, nopaline synthase terminator; P35S, CaMV 35S promoter; RB, T-region right border. (**B**) Somatic embryogenic callus derived from *PgDDS*-transformed roots of *A. elata*. Scale bar = 1 cm. (**C**) The presence of *PgDDS* in the transgenic *A. elata* calli was analyzed by a polymerase chain reaction. M, DNA size marker; P, positive control; WT, untransformed wild-type; lines 1–6, 8–24, and 26–28 are the transgenic calli showing the amplification of the *PgDDS* target gene; CK^−^, negative control. (**D**) Relative expression of the introduced *PgDDS* gene in the transgenic *A. elata* callus lines by quantitative real-time PCR arranged from lowest to highest. I, II, and III are low, intermediate, and high levels of *PgDDS* expression, respectively. Results are mean ± standard error of the mean (SEM).

*Agrobacterium tumefaciens* GV3101 carrying the *PgDDS* gene construct in the T-DNA vector (Figure 2A) was used for the genetic transformation of *A. elata* roots, and we tested three different infection times (5, 10, and 15 min). After eight weeks on a kanamycin selection culture medium, somatic embryogenic calli developed from the roots (Figure 2B), and the highest transformation rate was 27.14% after 10 min of infection (Table 1). A total of 26 independent kanamycin-resistant calli (L1–28 without L7 and L25) were obtained, and all 26 were then sub-cultured to allow them to proliferate. The 26 independent somatic embryogenic callus lines were confirmed to contain the *PgDDS* gene through a polymerase chain reaction (PCR) analysis of genomic DNA. No *PgDDS* gene fragment was amplified from wild-type (WT) *A. elata* DNA (Figure 2C).

Quantitative real-time PCR (qRT-PCR) was used to detect the relative transcription levels of the *PgDDS* gene in the 26 transgenic callus lines shown in Figure 2D. The *PgDDS* gene was found to be expressed in all 26 callus lines but not in the WT plants, and the expression levels differed among the lines, with the highest level of transcription detected in line L27.

### 2.2. Regeneration of Transgenic Plants and Phenotypic Observations

**Figure 3:**
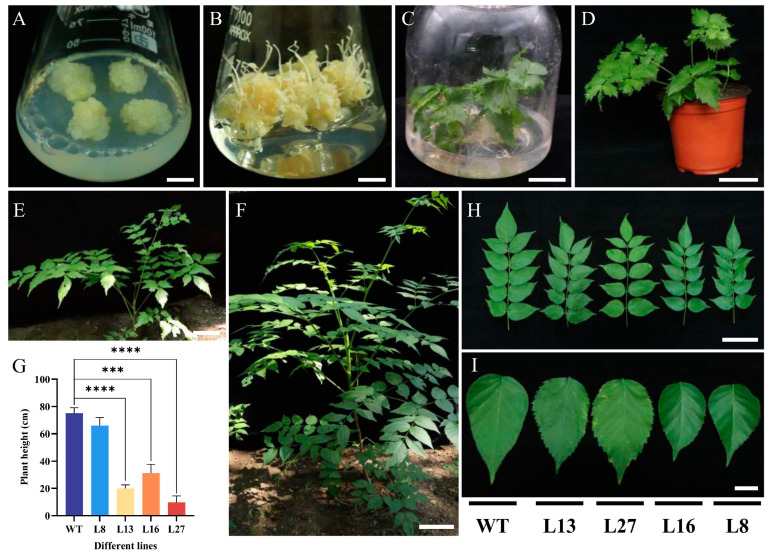
Regeneration and phenotypes of transgenic *A. elata* plants. (**A**) Transgenic callus proliferation. (**B**) Differentiation of transformed callus tissue into somatic embryo plantlets. (**C**) Transformed plantlets cultured in a plastic bottle. (**D**) Transgenic plants grown in soil for eight weeks. (**E**,**F**) Two-year old transgenic plant (**E**) and WT plant (**F**) grown in the experimental field. (**G**) Plant heights of two-year-old plants of the WT and the four transgenic lines of *A. elata*. Data shown are the means ± SEM. Statistical analysis was carried out by an ANOVA. The asterisks indicate the significant differences in plant height between the WT and the *PgDDS*-expressing transgenic lines. *** *p* < 0.001; **** *p* < 0.0001. (**H**,**I**) Leaf (**H**) and leaflet (**I**) phenotypes of representative plants of the WT and the four transgenic lines (L13, L27, L16, L8) of *A. elata*. Scale bars: (**A**), 1 cm; (**B**), 1 cm; (**C**), 2 cm; (**D**), 5 cm; (**E**), 10 cm; (**F**), 15 cm; (**H**), 10 cm; (**I**), 2 cm.

Based on the expression levels of *PgDDS* in the transgenic *A. elata* callus tissue, we selected four of the transgenic callus lines to regenerate plantlets through somatic embryogenesis. The selected lines were L27, with the highest level of *PgDDS* expression, L13 and L16, with low expression levels, and L8, with intermediate expression. The calli (Figure 3A) were cultured on a differentiation medium to induce somatic embryo formation, and after 4 weeks, the emerging shoots (Figure 3B) were transferred to a woody plant medium (WPM) [14] in plastic bottles (Figure 3C). After another 4 weeks of culture, the plant lines were transferred to small pots containing soil (Figure 3D). Two months later, the young plants were transplanted to a test field at our university. The growth of the transgenic plants was found to be slower than that of WT plants over a two-year period (Figure 3E,F). Except for the L8 plants, the plant heights of the L13 (19.93 cm), L16 (31.23 cm), and L27 (9.63 cm) plants were significantly reduced compared with the WT plants (74.97 cm), with the L27 plants being the shortest (9.63 cm) at only 12.85% the height of WT plants (Figure 3G). In addition, we also observed changes in leaf shape and size, and the serration of the leaf margins differed between the transgenic plants and the WT plants. The leaves of the transgenic plants exhibited larger leaf margin serrations compared with the WT plants. These differences were most pronounced in the L13 and L27 line plants (Figure 3H,I).

### 2.3. Expression of Key Enzyme Genes for Triterpene Synthesis in Transgenic Plant Leaves

**Figure 4:**
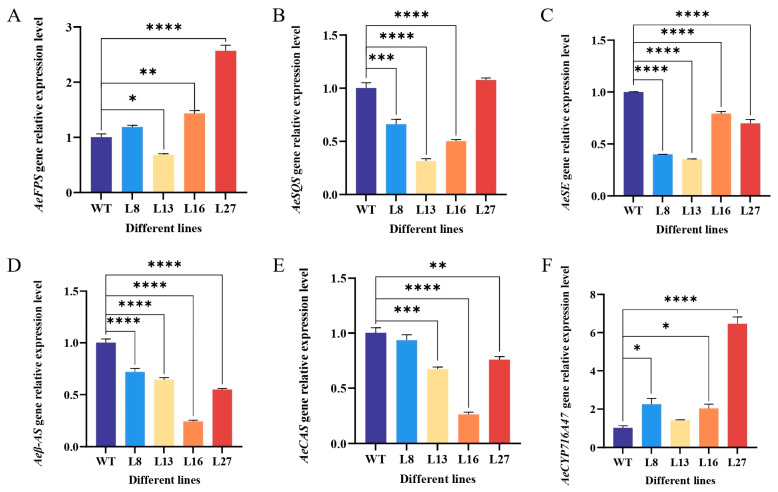
The influence of heterologous *PgDDS* expression on the expression of six key triterpene synthesis enzyme genes in transgenic *A. elata* plants. (**A**) *A. elata* farnesyl diphosphate synthase (*AeFPS*), (**B**) *A. elata* squalene synthase (*AeSQS*), (**C**) *A. elata* squalene epoxidase (*AeSE*), (**D**) *A. elata* β-amyrin synthase (*Aeβ-AS*), (**E**) *A. elata* cycloartenol synthase (*AeCSA*), and (**F**) *A. elata* protopanaxadiol synthase (*AeCYP716A47*). Data shown are the means ± SEM. Statistical analysis was carried out by an ANOVA. Asterisks indicate the statistical significance of gene expression differences between WT plants and the four transgenic lines expressing *PgDDS*. * *p* < 0.05; ** *p* < 0.01; *** *p* < 0.001; **** *p* < 0.0001.

We next investigated whether *PgDDS* expression affected the key enzyme genes in the mevalonic acid (MVA) pathway for triterpene synthesis in transgenic *A. elata*. The expression levels of *AeFPS*, *AeSQS*, *AeSE*, *Aeβ-AS*, *AeCAS*, and *AeCYP716A47* were measured in the leaves of the four transgenic plant lines using qRT-PCR (Figure 4). *AeFPS* expression was upregulated in the L8, L16, and L27 plants and downregulated in the L13 plants. The L8, L16, and L27 plants expressed significantly lower levels of *AeSQS* than the WT plants, but they were slightly higher in the L27 plants, although the differences were not significant compared with the WT plants. *AeSE*, *Aeβ-AS*, and *AeCAS* were down-regulated in all four transgenic lines. The expression of the *AeCYP716A47* gene was higher in the plants of the four transgenic lines compared with the WT plants.

### 2.4. UPLC-MS/MS-Based Quantitative Metabolomic Analyses of the Leaves of Transgenic Plants

To better understand the dynamics of the relevant terpene metabolites in *A. elata* plants expressing the *P. ginseng PgDDS* gene, we analyzed 70% methanol extracts made from the leaves of the transgenic plants and the WT plants using UPLC-MS/MS. Based on the significant differences between the transgenic and WT plants in terms of the expression of key enzyme-encoding genes involved in triterpene synthesis, plant height, and leaf shape, the L13 and L27 plants were selected for the terpene metabolome analysis. In the Metware metabolite database (MWDB), qualitative analyses reflect second-order spectral information. Data from mass spectra were processed using Analyst 1.6.3 software. Total ion chromatography (TIC) represents a continuous analysis of the total intensity sum of all ions in a mass spectrum over time. In a typical TIC of a quality control (QC) sample, the total ion flow curve overlaps, and the retention time and peak intensity are the same (Supplementary Figure S1A,B), indicating that the results were reliable. Supplementary Figure S1C,D show the multi-peak detection plot of metabolites acquired in MRM mode.

**Figure 5:**
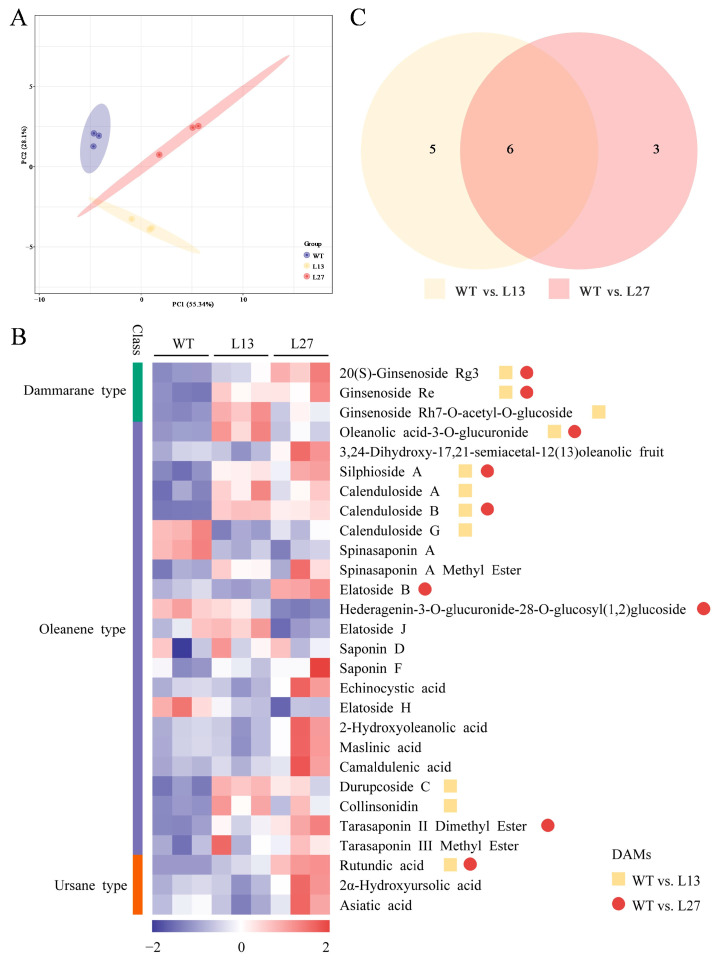
Terpenoid metabolomes of WT and transgenic (L13 and L27) plants. (**A**) Principal component analysis (PCA) of differentially accumulated terpene metabolites between WT *A. elata* plants and transgenic L27 and L13 line plants. Score plots of principal components 1 (PC1) and PC2 represent high cohesion within groups and good separation among the three lines. The sampling groups are color-coded as follows: purple, WT; yellow, L13; and red, L27. (**B**) Heatmap of terpene metabolites (dammarane-type, oleanene-type, and ursane-type) from WT and transgenic (L13 and L27) plants. Relative abundances are shown as terpene metabolites from three individual plants of each line (WT, L13, and L27). Columns represent biological replicates for each treatment. Yellow squares, differentially accumulated metabolites (DAMs) between WT and L13 plants; red circles, DAMs between WT and L27 plants. (**C**) Venn diagram showing the number of shared and unique DAMs in the WT vs. L13 and WT vs. L27 comparisons.

A principal component analysis (PCA) was performed on the samples in order to gain a preliminary understanding of the total metabolic differences between the samples in each group and the degree of variation between the samples within the groups. The first two principal components (PCs) could separate nine samples clearly, accounting for 83.44% of the total variability. PC1 contributed to 55.34% of the variability, while PC2 accounted for 28.1% (Figure 5A). The data suggested that the differences in terpene metabolites between the transgenic line plants (L13 and L27) and the WT plants were significant.

### 2.5. Identification of Differentially-Accumulated Metabolites

The data analysis from Section 2.4 is shown in Figure 5B. All the terpene metabolites belonged to the triterpenoid type in the leaves, and there were 28 compounds in the WT plants and the transgenic lines L13 and L27. Among them, three compounds were tetracyclic triterpenoids (dammarane-type), namely 20(S)-ginsenoside Rg3, ginsenoside Re, and ginsenoside Rh7-O-acetyl-O-glucoside, and the others were pentacyclic triterpenoids and included 22 oleanolic types and three ursane types. Differentially accumulated metabolites (DAMs) were identified using the fold-change (FC ≥ 2 or FC ≤ 0.5) and OPLS-DA model (variable importance in projection (VIP) value ≥1) data. Eleven DAMs (ten upregulated and one downregulated) were identified in the L13 vs. WT comparison (Figure 5B). These 11 DAMs were 20(S)-ginsenoside Rg3, ginsenoside Re, ginsenoside Rh7-O-acetyl-O-glucoside, oleanolic acid-3-O-glucuronide, collinsonidin, calenduloside A, silphioside A, durupcoside C, calenduloside B, rutundic acid, and calenduloside G. The following nine DAMs (eight upregulated and one downregulated) were identified in the L27 vs. WT comparison: 20(S)-ginsenoside Rg3, ginsenoside Re, oleanolic acid-3-O-glucuronide, silphioside A, calenduloside B, tarasaponin II dimethyl ester, elatoside B, rutundic acid, and hederagenin-3-O-glucuronide-28-O-glucosyl(1,2)glucoside (Figure 5B).

The Venn diagram showed that some DAMs were shared between the WT vs. L13 and WT vs. L27 comparisons (Figure 5C). Among them, six DAMs are worth mentioning, and the two dammarane-type saponins, Rg3 and Re, were upregulated in the transgenic lines compared with the WT plants (Figure 5B). Three other oleanolic-type saponins (oleanolic acid-3-O-glucuronide, silphioside A, and calenduloside B) and one ursane-type saponin (rutundic acid) were also obviously upregulated in the leaves of the transgenic L13 and L27 line plants compared with the WT plants. In addition, the levels of collinsonidin, calenduloside A, durupcoside C, and ginsenoside Rh7-O-acetyl-O-glucoside were only upregulated in the L13 plants; tarasaponin II dimethyl ester and elatoside B were only upregulated in the L27 plants, and calenduloside G and hederagenin-3-O glucuronide-28-O-glucosyl(1,2)glucoside were downregulated in the L13 and L27 plants, respectively. These data imply that the expression of the *PgDDS* gene in the transgenic plants not only upregulated the synthesis of tetracyclic dammarane-type triterpenoid saponins but also upregulated some pentacyclic oleanane-type triterpenoid saponins.

### 2.6. High-Performance Liquid Chromatography (HPLC) Analysis of the Rg3 and Re Contents in the Leaves of L13 and L27 Plants

**Figure 6:**
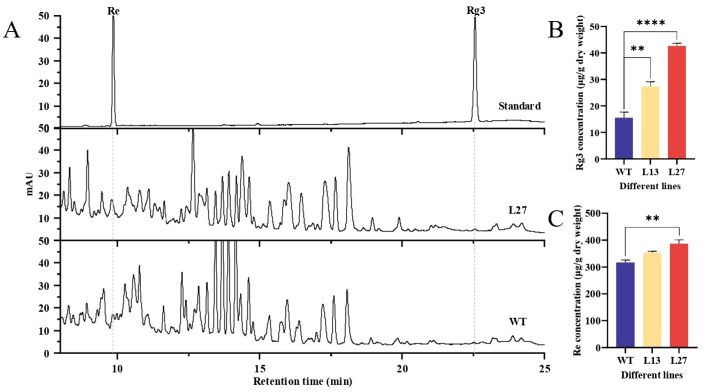
High-performance liquid chromatography (HPLC) analysis of extracts from L13, L27, and WT plants. (**A**) Examples of HPLC chromatograms of extracts from a WT *A. elata* plant, a plant of the L27 transgenic line, and known Rg3 and Re standards. (**B**,**C**) Quantifications of Rg3 and Re contents in the leaves of L13, L27, and WT plants. Results are mean ± SEM. Statistical analysis was performed by an ANOVA, and asterisks indicate significant differences between transgenic and WT plants. ** *p* < 0.01; **** *p* < 0.0001.

Based on the results of the terpene metabolomic analysis, the expression of *PgDDS* increased the contents of the dammarane-type saponins Rg3 and Re in *A. elata*. We selected plants of the transgenic lines L13 and L27 and WT plants for analysis of their Rg3 and Re contents using HPLC. The HPLC chromatograms of Rg3 and Re for the transgenic L27 and WT plants and the standards are shown in Figure 6A. The contents of Rg3 and Re were calculated using the linear equations Y = 3.193 X + 0.8241 (R^2^ = 0.999) and Y = 2.3592 X + 2.0104 (R^2^ = 0.9994), respectively, in which Y represents the peak area and X represents the content (μg/mL). The contents of Rg3 and Re in the transgenic L13 and L27 plants were higher than in WT plants, which is consistent with the results of the metabolomic analysis. The Rg3 content in the leaves of the transgenic line L27 plants was 42.64 μg/g, which was very significantly different than that in the WT plants (15.48 μg/g). The Re content was 386.81 μg/g in the L27 plants, which was also significantly different from that in the WT plants (316.96 μg/g) (Figure 6B,C).

## 3. Discussion

In this study, the key enzyme gene *PgDDS* for tetracyclic triterpenoid biosynthesis in *P. ginseng* was introduced into the genome of *A. elata*, a medicinal plant from the same botanical family (Araliaceae), using *A. tumefaciens*-mediated transformation. The heterologous expression of *PgDDS* changed not only the growth and leaf size/shape of the transgenic *A. elata* plants but also the contents of tetracyclic and pentacyclic terpenoids in the leaves.

In this study, a highly efficient genetic transformation system was established for *A. elata*. At present, only one study has reported the use of the *Agrobacterium rhizogenes*-mediated transformation of petioles and root segments to obtain transgenic *A. elata* hairy roots and regenerated plants [15]. The highest transformation rate of 27.14% was achieved with an infection time of 10 min. This efficient genetic transformation system is important for the study of saponin metabolism and regulation in species in the Araliaceae family.

The heterologous expression of *PgDDS* affects the expression of related genes in plants (Figure 4). The expression of *PgDDS* upstream and downstream genes in transgenic plants was examined, and the results showed that the expression levels of *AeSQS* and *AeSE* decreased, but the expression levels of *AeFPS* and *AeCYP716A47* increased. Meanwhile, the expression levels of *FPS*, *SQS*, *SE*, and *DDS* increased in a *Panax notoginseng* cell overexpressing *Pjβ-AS* [16], which may be caused by the differences in metabolism between the cells and plantlets. PgDDS shares the same precursor, 2,3-oxidosqualene, with the other oxidosqualene cyclases (OSCs) in the saponin biosynthesis pathway (e.g., β-AS and CAS) (Figure 1), and because of the effect of expressing *PgDDS* in *A. elata*, the expression of *AeCAS* and *Aeβ-AS* was reduced in the transgenic lines in our study (Figure 4). And the *CAS* gene encodes the key enzyme for the biosynthesis of phytosterols in plants and is relevant to growth and development in *Arabidopsis thaliana* and tobacco plants [17,18]. In *A. thaliana*, one type of phytosterol, campesterol, was shown to be a precursor of brassinolide (BL), which affects stem elongation and development [19,20,21]. The application of the bioactive brassinosteroid (BR) BL restores normal growth to BR biosynthesis mutants in tomato (*Lycopersicon esculentum*), pea (*Pisum sativum*), rice (*Oryza sativa*), and maize (*Zea mays*) [22]. Therefore, how the expression of *PgDDS* in *A. elata* influenced the expression of *CAS* and BL biosynthesis on growth will be studied further in our future research.

The analyses of the terpene metabolites in the leaves of the WT, L13, and L27 plants showed that the 28 triterpenoids identified [tetracyclic triterpenoids and pentacyclic triterpenoids (oleanane-type and ursane-type)], were almost the same (besides rutundic acid) between the WT and transgenic lines and that the *PgDDS* expression in the L13 and L27 lines did not result in the production of other terpenes, which is thought to be beneficial in a plant consumed for medicinal purposes. The analysis showed that the ursane-type triterpenoids identified in our study did not belong to the five classes of triterpenoid structural skeletons in *A. elata* previously reported by Zhang et al. [5] and according to other information on related biosynthesis enzymes also did not be checked [23,24]. The overexpression of the *PgDDS* gene in *A. elata* provided useful material for investigating changes in metabolites, and more metabolites may be detected by expanded metabolomics analysis in future research. Additionally, the increased accumulation of metabolites may improve the medicinal and edible qualities of *A. elata*, but assessing its impacts on ecology and the environment will be carried out in future work. 

A pathway for the biosynthesis of tetracyclic terpenoids is present in *A. elata*, and Re has also been identified [5,9]. In our study, *PgDDS*, the first key enzyme-encoding gene involved in tetracyclic triterpenoid biosynthesis in *P. ginseng*, was overexpressed in *A. elata*, and the terpene metabolomic analysis of the leaves showed that the contents of the rare and medicinally valuable saponins Rg3 and Re were notably higher in the plants of the transgenic lines L13 and L27 than the WT plants. However, the direct product of *PgDDS*, dammarenediol-II (DD), was not detected in the metabolome, probably because DD is an intermediate product of ginsenoside synthesis that is rapidly transformed by UDP-dependent glycosyltransferase (UGT) into final ginsenoside products. Similarly, the key enzyme-encoding gene *Pjβ-AS* involved in pentacyclic triterpenoid biosynthesis in *Panax japonicus* was overexpressed in *P. notoginseng*, and only chikusetsusaponin IV and chikusetsusaponin IVa could be detected, whereas the direct product of *Pjβ-AS*, β-Amyrin, was not detected [16]. However, in *PgDDS*-expressing transgenic lines of rice and tobacco, the heterologous expression products DD, protopanaxadiol (PPD), and protopanaxatriol (PPT) could be detected [12,13,25]. Furthermore, we could not detect the expression of the endogenous *AeDDS* gene in either the WT plants or the transgenic lines. The expression of this endogenous gene was very low in the leaves, which could explain why we were unable to detect it. The analysis of the genome sequence of *A. elata* showed that there is a deletion in the *AeDDS* gene sequence that could lead to its weak function [24].

## 4. Materials and Methods

### 4.1. Construction of Plant Expression Vectors and Plant Transformation

The total RNA of *P. ginseng* was isolated from adventitious roots using the CTAB method. Complementary DNA (cDNA) was synthesized using the PrimeScript™ RT reagent kit with a gDNA eraser (Takara Bio Inc., Dalian, China). The DNA sequence of the open reading frame (ORF) of the *PgDDS* gene (GenBank accession number: AB122080.1) was obtained from cDNAs amplified using the primer pair 5′-ATCTCTAGAATGTGGAAGCTGAAGGTTGCTC-3′ and 5′-ATCGAGCTCTTAAATTTTGAGCTGCTGGTGC-3′. The gene was cloned using the pEASY-Blunt vector (TransGen Biotech, Beijing, China) and transferred into the destination vector pROKII under the control of the CaMV 35S promoter. A freeze–thaw procedure was then used to introduce pROKII-*PgDDS* into the *A. tumefaciens* strain GV3101.

In our previous research, a plant regeneration system involving somatic embryogenesis and a root explant transformation system were established for *A. elata* [1,26]. The roots from the somatic embryogenic plants were further pre-cultured on a Murashige and Skoog (MS) [27] callus induction medium containing 0.5 mg/L of 2,4-dichlorophenoxyacetic acid (2,4-D) and 3% sucrose. The pre-cultured explants were immersed in a cell suspension of *A. tumefaciens* GV3101 containing pROKII-*PgDDS* for 5–15 min. After three days of co-culture on the callus induction medium, the root segments were transferred to a somatic embryogenic callus induction medium containing 200 mg/L of timentin and 50 mg/L of kanamycin. After 8 weeks, kanamycin-resistant calli were obtained.

### 4.2. PCR Identification of Transgenic Callus Lines

Genomic DNA was isolated from the callus tissue of the putative transformants and WT *A. elata* using a CTAB-based method. Plasmid DNA from *E.coli* was isolated using the Plasmid Mini Extraction Kit (Omega, Norcross, GA, USA). The *PgDDS* gene was detected in genomic DNA by PCR using the primer pair pROKII-F: 5′-CGCACAATCCCACTATCCTT-3′ and pROKII-R: 5′-AAGACCGGCAACAGGATTC-3′.

### 4.3. Transgenic Plantlets

To induce somatic embryogenesis and shoot development, the identified transgenic callus lines were transferred to somatic embryogenesis conditions on a differentiation medium [26]. After 4 weeks, the somatic embryo plantlets were transferred to a woody plant medium (WPM) [14] supplemented with 20 g/L of sucrose. After 4 weeks of culture, the regenerated transgenic plants were transferred to pots containing soil, and 8 weeks later, they were transferred to the experimental field. After two years in the field, the heights of the plants from the soil surface to the apical buds were measured, and all lines had at least three replicates.

### 4.4. qRT-PCR Analysis

Total RNA was isolated from the callus tissue and leaves of the regenerated plants using a Universal Plant Total RNA Extraction Kit (Spin-column; Bioteke Corporation, Beijing, China). Total RNA was reverse-transcribed into first-strand cDNA using the PrimeScript RT reagent kit with a gDNA eraser (Takara Bio Inc., Dalian, China). TransStart Green qPCR SuperMix (TransGen Biotech, Beijing, China) and the ABI 7500 Real-Time PCR system (Applied Biosystems, Forster City, CA, USA) were used to determine the relative expression of *PgDDS*, *AeFPS*, *AeSQS*, *AeSE*, *Aeβ-AS*, *AeCAS*, and *AeCYP716A47* by qRT-PCR. The qRT-PCR amplification conditions were 95 °C for 5 min, followed by 40 cycles of 95 °C for 5 s and 60 °C for 34 s. *GAPDH* served as the internal reference for the normalization of gene expression. The qRT-PCR primer pairs are given in Supplementary Table S1. All qRT-PCRs were performed at least three times.

### 4.5. Metabolite Profiling Using UPLC-MS/MS

The leaves of the two-year-old plants described in Section 4.3 were used for the analysis of terpene metabolites. The leaves were first freeze-dried in a lyophilizer (Scientz-100F, Ningbo, China) and then ground using a mixer mill (MM 400, Retsch, Haan, Germany) with zirconia beads for 1.5 min at 30 Hz. Next, 0.1 g of lyophilized powder of *A. elata* leaves was dissolved in 1.2 mL of 70% methanol, and the mixture was vortex-mixed for 30 s every 30 min for a total of six times and stored at 4 °C overnight. The extracts were centrifuged at 12,000 rpm for 10 min, filtered through a membrane (SCAA-104, 0.22 μm pore size; ANPEL, Shanghai, China, http://www.anpel.com.cn/), and subjected to a UPLC-MS/MS analysis.

The *A. elata* leaf extracts were analyzed using an UPLC-ESI-MS/MS system (UPLC, Shimadzu Nexera X2, Shanghai, China, www.shimadzu.com.cn/; MS, Applied Biosystems 4500Q TRAP, www.appliedbiosystems.com.cn/). The analytical conditions were as follows: UPLC; column, Agilent SB-C18, Santa Clara, CA, USA, (1.8 μm, 2.1 mm × 100 mm); the mobile phase consisted of solvent A, 0.1% formic acid in water, and solvent B, acetonitrile containing 0.1% formic acid. An elution gradient program with 95% A and 5% B was used for sample elution. A linear gradient of 5% A and 95% B was employed for 9 min; the composition of 5% A and 95% B was held from 9 to 10 min, then changed to 95% A and 5.0% B within 1.10 min and held from 11.10 to 14 min. The flow rate was 0.35 mL/min, the temperature of the column was set at 40 °C, and the injection volume was set to 4 μL. The effluent was transported to an ESI- triple quadrupole-linear ion trap (QTRAP)-MS.

Linear ion trap (LIT) and triple-quadrupole (QQQ) scans were acquired from a triple quadrupole-linear ion trap mass spectrometer (Q TRAP) AB4500 Q TRAP UPLC/MS/MS System. The system was equipped with an ESI turbo ion-spray interface, operated in positive and negative ion mode, and controlled using Analyst 1.6.3 software (AB Sciex, Framingham, MA, USA). The ESI source operation parameters were as follows: ion source, turbo spray; source temperature, 550 °C; ion spray voltage [28], 5500 V (positive ion mode) and −4500 V (negative ion mode); ion source, gas I (GSI), gas II (GSII), and curtain gas (CUR); pressures, 50, 60, and 25.0 psi, respectively; and collision-activated dissociation (CAD), high. In addition, 10 μmol/L and 100 μmol/L of polypropylene glycol solutions were used to tune the instrument and perform mass calibration in the QQQ and LIT modes, respectively. QQQ scans were acquired in multiple-reaction monitoring (MRM) experiment mode, and the nitrogen used as collision gas was set to medium. Decluttering potential (DP) and collision energy (CE) for individual MRM transitions were completed by further optimization of the DP and CE. A specific set of MRM transitions was monitored for each period based on the terpene metabolites eluted within this period. All terpene metabolites were identified and annotated using the Metware database.

### 4.6. HPLC Analysis of Rg3 and Re in Leaves of Transgenic Plants

The accumulation of Rg3 and Re was measured by HPLC using the method described by Guo et al. [29]. The HPLC analysis was performed on an Agilent 1100 with a C18 column (250 mm × 4.6 mm, 5 μm) and a gradient elution with acetonitrile/0.1% phosphoric acid solution at 20/80 (*v*/*v*) for 20 min, 60/40 for 15 min, and 20/80 for 20 min. The flow rate was set at 1 mL/min, the injection volume was 10 μL, and the sample peaks were detected by UV absorption at 203 nm. The known standards, ginsenoside Rg3 (Batch No. 14197-60-5) and ginsenoside Re (Batch No. 52286-59-6), were obtained from the Shanghai Yuanye Biotechnology Company Limited (Shanghai, China).

### 4.7. Statistical Analyses

The PCA and heatmap analyses were performed using the Metware Cloud, a free online platform for data analysis (https://cloud.metware.cn). Significantly altered metabolites between groups were determined based on a VIP value ≥1 and an absolute Log_2_FC (fold change) ≥1, and the VIP values of each metabolite were extracted from the OPLS-DA results. Data were Log2-transformed and mean-centered before OPLS-DA. A permutation test (200 permutations) was performed to avoid overfitting. All experiments included at least three biological replicates.

## 5. Conclusions

In this study, we established a stable and efficient genetic transformation system using *A. tumefaciens* GV3101 to introduce the *PgDDS* gene from *P. ginseng* into the *A. elata* genome and obtained stable transgenic plants. The transgenic plants were shorter in stature than the WT plants, and there were obvious differences in the shapes and sizes of the leaflets and the leaf margin serration. Also, the *PgDDS* transformation in *A. elata* changed the relative expression of genes encoding other enzymes in the terpenoid biosynthesis pathway as well as the contents of some tetracyclic and pentacyclic triterpenoid compounds. In particular, the levels of the tetracyclic triterpenoid saponins Rg3 and Re were significantly increased in the plants of the transgenic line L27. We expect that the transgenic *A. elata* plants with higher Rg3 and Re contents produced in this study will have increased medicinal benefits for consumers.

## Figures and Tables

**Table 1:** Effect of infection time on callus formation rate in *Aralia elata* roots transformed with *Agrobacterium tumefaciens* GV3101.

Infection Time (min)	No. of Explants	No. of Callus-Forming Explants	% of Transformed Callus Explants
5	70	1	1.43
10	70	19	27.14
15	70	6	8.57

## Data Availability

Data are contained within the article and Supplementary Materials.

## Supplementary Materials

The following supporting information can be downloaded at https://www.mdpi.com/article/10.3390/ijms25031945/s1.
