# Supplementary material for: PgDDS Changes the Plant Growth of Transgenic Aralia elata and Improves the Production of Re and Rg3 in Its Leaves
Source: Int J Mol Sci. 2024 Feb 5;25(3):1945. doi: 10.3390/ijms25031945 (PMC10856007; doi:10.3390/ijms25031945)
Supplement: Supplementary file 1 [file ijms-25-01945-s001.zip › ijms-2805014-Supplementary Figure S1.pptx]

## Slide 1
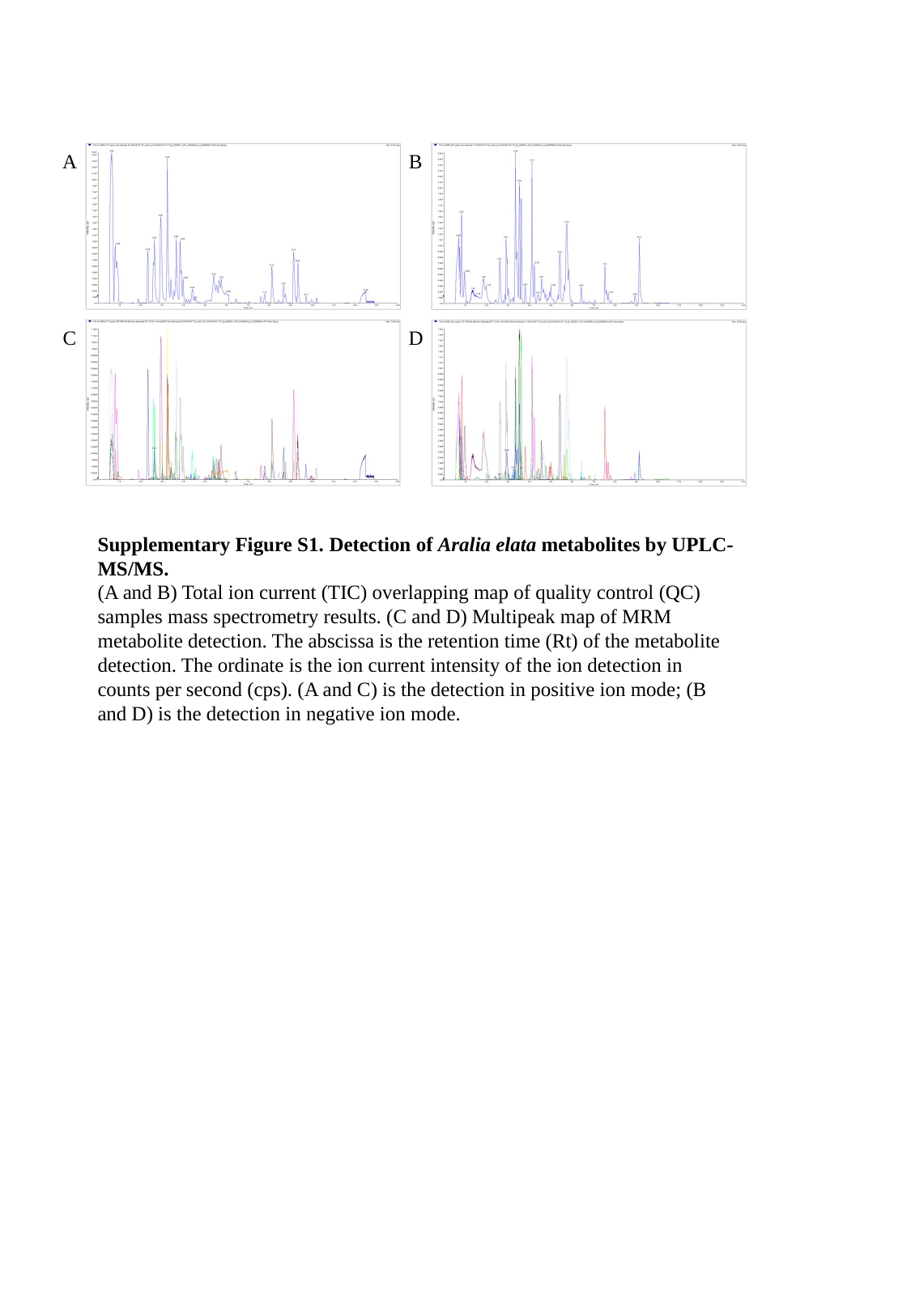

A
B
C
D
Supplementary Figure S1. Detection of Aralia elata metabolites by UPLC-MS/MS.
(A and B) Total ion current (TIC) overlapping map of quality control (QC) samples mass spectrometry results. (C and D) Multipeak map of MRM metabolite detection. The abscissa is the retention time (Rt) of the metabolite detection. The ordinate is the ion current intensity of the ion detection in counts per second (cps). (A and C) is the detection in positive ion mode; (B and D) is the detection in negative ion mode.
